# Thermal Response-Based Evaluation of Non-Ablative Fractional Er:Glass Laser Therapy for Scar Management: A Retrospective Observational Study with Forward-Looking Infrared (FLIR) Monitoring

**DOI:** 10.3390/jcm14248910

**Published:** 2025-12-17

**Authors:** Ha Jong Nam, Se Young Kim, Hwan Jun Choi

**Affiliations:** 1 Department of Plastic and Reconstructive Surgery, Soonchunhyang University Gumi Hospital, Gumi 39371, Republic of Korea; 125039@schmc.ac.kr (H.J.N.); soulloop@naver.com (S.Y.K.); 2 Department of Plastic and Reconstructive Surgery, Soonchunhyang University Cheonan Hospital, Cheonan 31151, Republic of Korea

**Keywords:** laser therapy, non-ablative laser treatment, cicatrix, regeneration, thermography

## Abstract

**Background/Objectives:** Non-ablative fractional lasers are widely used for scar remodeling, yet treatment parameters are often selected empirically, and thermal thresholds for consistent outcomes remain undefined. This study explored whether forward-looking infrared (FLIR) thermography can estimate laser-induced surface temperature changes during 1550 nm Er:Glass laser therapy and examined the association between post-treatment temperature elevation and early clinical improvement. **Methods:** A retrospective analysis was conducted on patients treated with fractional Er:Glass laser for post-surgical or traumatic scars. Skin temperature was recorded using FLIR C5 imaging at baseline (T_0_), after topical anesthesia (T_1_), and immediately post-treatment (T_2_). The temperature change (ΔT_2_) was calculated as T_2_ − T_0_. Clinical outcomes were assessed one month after treatment using standardized digital photographs and Vancouver Scar Scale (VSS) scores. Safety data were collected from post-procedure observations and patient reports. **Results:** Mean surface temperature increased from 32.4 ± 0.9 °C at T_0_ to 33.7 ± 0.7 °C at T_2_ (ΔT_2_ = +1.3 ± 0.6 °C, *p* < 0.001). Hypertrophic scars showed higher ΔT_2_ values than linear scars (*p* = 0.02). A moderate temperature elevation was modestly associated with early VSS improvement (*r* = 0.42, *p* = 0.003). Representative cases with ΔT_2_ values around 1.5–2.5 °C exhibited favorable short-term changes in texture and pigmentation. No adverse events were observed during follow-up. **Conclusions:** Real-time FLIR thermography may provide a non-invasive method to indirectly assess surface thermal response during non-ablative fractional treatment. A moderate temperature increase may be associated with an exploratory thermal response range linked to early clinical improvement, but the findings are preliminary. Further prospective, controlled studies with standardized treatment parameters and longer follow-up are required to clarify whether ΔT_2_ has clinical relevance as a physiologic parameter for temperature-based assessment in scar management.

## 1. Introduction

The scarring resulting from trauma, surgery, or burns can profoundly affect physical appearance and psychosocial well-being. Accordingly, effective scar management remains a critical challenge in reconstructive and aesthetic medicine [1,2]. Among the available treatment modalities, fractional laser therapy has emerged as one of the most effective minimally invasive options for improving scar texture, color, and pliability [3,4,5].

Fractional laser systems are broadly categorized into ablative and non-ablative types. Ablative lasers, such as carbon dioxide (CO_2_) and erbium-doped yttrium aluminum garnet (Er:YAG) devices, vaporize the epidermis to induce dermal remodeling but are associated with prolonged recovery times and an increased risk of adverse effects [6,7]. In contrast, non-ablative fractional lasers, including erbium-doped glass (Er:Glass, 1550 nm) systems, selectively target dermal microthermal columns while preserving the epidermal barrier [4]. This mechanism produces controlled thermal injury that stimulates neocollagenesis with minimal downtime, making non-ablative approaches particularly advantageous for Asian skin types, which are more prone to post-inflammatory hyperpigmentation [8,9]. Non-ablative fractional photothermolysis is known to generate controlled microthermal zones within the dermis, where collagen denaturation, thermal diffusion, and subsequent extracellular matrix remodeling occur in a heat-dependent manner [10]. These thermal dynamics form the biological foundation for temperature-guided approaches in non-ablative laser therapy.

Despite their widespread clinical use, the intraprocedural thermal dynamics of non-ablative fractional lasers remain poorly characterized [10,11]. Early experimental work demonstrated that fractional photothermolysis generates sharply demarcated microthermal zones with predictable patterns of dermal coagulation and epidermal sparing, providing mechanistic insight into heat-dependent tissue remodeling [12]. Building on these foundational observations, current treatment protocols still rely largely on empirically determined energy settings, without real-time feedback on cutaneous temperature or tissue response [4,13]. Because both treatment efficacy and safety are closely dependent on the degree of thermal diffusion within the dermis, an objective method for monitoring temperature changes during treatment could significantly enhance treatment precision and patient safety [10,14,15]. Previous optical and dermatologic studies have emphasized that the extent of intradermal heating is a critical determinant of both efficacy and safety during fractional laser procedures, underscoring the need for objective real-time monitoring [14,16]. Thermal monitoring has increasingly been recognized as an important component of safe and effective energy-based treatment, as it provides physiologic information that cannot be obtained through visual assessment alone. Several investigations have demonstrated the utility of infrared thermography for visualizing cutaneous heat distribution during energy-based procedures, highlighting its potential as a real-time adjunct to guide energy delivery. Despite these advances, temperature-guided optimization remains underexplored in non-ablative fractional laser therapy, reinforcing the need for systematic evaluation of intraprocedural thermal behavior.

Forward-looking infrared (FLIR) thermography enables non-contact, high-resolution measurement of skin-surface temperature distribution in real time [16]. Initially developed for industrial and military purposes, FLIR imaging has since been applied in various medical settings, including flap perfusion monitoring, burn-depth assessment, and evaluation of peripheral circulation [14,17]. Medical thermography has additionally been explored for characterizing cutaneous thermal gradients, estimating subsurface perfusion, and evaluating temperature-dependent tissue responses, supporting its relevance as a physiological monitoring tool during energy-based dermatologic procedures [17]. Infrared thermography has also been validated in clinical wound assessment, with systematic reviews demonstrating its utility for estimating burn-depth progression and healing potential [18]. These data further support the relevance of thermal imaging as a physiologic monitoring tool in dermatologic procedures. Its incorporation into laser dermatology offers the potential to dynamically visualize thermal profiles, allowing clinicians to maintain a therapeutic temperature range that promotes collagen remodeling while avoiding excessive thermal injury [10,15].

Therefore, in this study, we aimed to quantify skin-surface temperature changes during Er:Glass non-ablative fractional laser treatment using real-time FLIR thermography and to correlate these thermal dynamics with early clinical outcomes. By exploring whether a clinically meaningful “thermal window” may be identified as an indicator of safe and effective treatment response, we sought to provide preliminary, evidence-based insight into temperature-guided non-ablative fractional laser therapy in scar management.

## 2. Materials and Methods

### 2.1. Study Design

This retrospective clinical study was conducted in the Department of Plastic and Reconstructive Surgery at Soonchunhyang University Hospital, Korea. Medical records of patients who underwent non-ablative fractional Er:Glass laser treatment for scar management between March 2023 and February 2024 were reviewed. The study protocol received approval from the Institutional Review Board of Soonchunhyang University Hospital (IRB No. 2025-03), and all procedures adhered to the ethical principles of the Declaration of Helsinki.

### 2.2. Patient Selection and Eligibility Criteria

A total of 61 patients who received non-ablative fractional Er:Glass laser treatment for surgical, traumatic, or burn scars during the study period were screened. After applying inclusion and exclusion criteria, 55 patients were included in the final analysis. Patients presented with scars located in different anatomical regions (face, trunk, and extremities). Anatomical site was documented for all cases and incorporated as a covariate in subsequent statistical analyses to account for site-related variability.

Inclusion criteria were: (1) clinically stable scars older than six months, (2) no additional scar treatment within the preceding three months, and (3) availability of complete thermal imaging and follow-up data for at least one month. Exclusion criteria were: (1) active infection or inflammation at the treatment site, (2) systemic corticosteroid or immunosuppressant use, (3) pregnancy or lactation, and (4) history of impaired wound healing or hypertrophic scarring unrelated to the treated lesion. Clinical records were independently reviewed by two investigators to confirm data completeness and consistency.

### 2.3. Laser Treatment Protocol

All patients received a single session of non-ablative fractional Er:Glass laser therapy (Sellas EVO, Dinona, Seoul, Republic of Korea; wavelength 1550 nm). A topical anesthetic cream containing 2.5% lidocaine and 2.5% prilocaine (EMLA^®^, AstraZeneca, Cambridge, UK) was applied evenly for 30 min and removed prior to irradiation.

Laser settings were adjusted based on scar type and anatomical location. Facial scars were typically treated at 2 mJ, whereas thicker or hypertrophic scars on the trunk or extremities received 2–4 mJ. Spot density was standardized at 100 pulses per area, and the laser was applied ten times in moving (continuous-scanning) mode with approximately 50% overlap. The beam pattern was set to an elliptical shape and adjusted according to the scar size and contour, extending slightly beyond the visible scar margin to ensure uniform dermal coverage.

**Figure 1:**
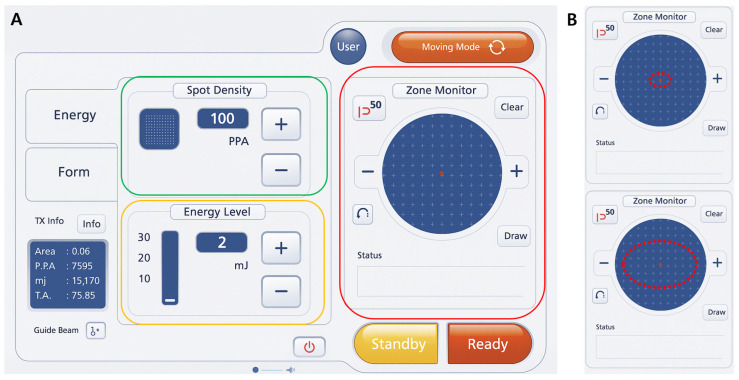
(**A**) User interface of the Er:Glass fractional laser system (Sellas EVO, Dinona, Republic of Korea). The green box indicates the spot density (100 PPA), the yellow box the energy level (2 mJ), and the red box the Zone Monitor used to define and visualize the treatment area in real time. (**B**) Customizable irradiation pattern displayed on the Zone Monitor. The elliptical beam shape was adjusted according to the scar size and contour to ensure uniform coverage while maintaining consistent round beam geometry.

Post-treatment care consisted of cooling with sterile saline-soaked gauze for five minutes, followed by application of a mild moisturizer and sunscreen. No occlusive dressing or adjunctive medication was used. A representative interface of the Er:Glass laser system is shown in Figure 1.

### 2.4. Thermal Imaging Acquisition and Calibration

Thermal imaging was performed using a handheld forward-looking infrared camera (FLIR C5, Teledyne FLIR, Wilsonville, OR, USA; thermal sensitivity ≤ 0.1 °C; resolution 160 × 120 pixels). Images were obtained at three time points: immediately before topical anesthesia (T_0_), after 30 min of anesthetic application (T_1_), and immediately post-treatment (T_2_). T_2_ images were obtained immediately after laser irradiation and prior to the 5 min saline cooling step to ensure that the measurement reflected the actual laser-induced temperature elevation. The camera was positioned 30 cm from the skin at a 90° angle. Room temperature (22–24 °C) and relative humidity (40–50%) were controlled to minimize environmental variability.

Skin emissivity was set to 0.98. Images were analyzed using FLIR Tools software (version 6.4; Teledyne FLIR, USA). A circular region of interest approximately 1 cm in diameter was placed at the scar center. Mean surface temperature was recorded, and post-treatment temperature change (ΔT_2_) was defined as T_2_ minus T_0_. We used T_0_ (pre-anesthesia baseline) as the reference point because topical anesthesia can transiently alter skin temperature through cooling effects and occlusion, making T_1_ an unreliable baseline for measuring true laser-induced thermal elevation. Therefore, ΔT_2_ = T_2_ − T_0_ was selected to more accurately reflect the physiologic thermal response to laser irradiation. All images were captured by a single operator, and discrepancies were jointly verified and resolved by consensus with a second investigator.

### 2.5. Clinical and Patient-Reported Outcome Measures

Clinical assessments were performed at baseline, one week, and one month post-treatment. Standardized digital photographs were taken under identical lighting and positioning conditions. Scar area was quantified using ImageJ software version 1.53 (National Institutes of Health, Bethesda, MD, USA), and percentage reduction from baseline was calculated.

Objective scar quality was assessed using the Vancouver Scar Scale (VSS), which evaluates pigmentation, vascularity, pliability, and height. Two independent board-certified plastic surgeons, blinded to treatment parameters, scored each case, and mean values were used for analysis.

Subjective metrics included pain measured by a 10-point visual analog scale (VAS) and patient satisfaction assessed on a 5-point Likert scale. All evaluations were conducted at the same scheduled follow-up visits.

### 2.6. Statistical Analysis

Statistical analyses were performed using SPSS software (version 26.0; IBM Corp., Armonk, NY, USA). Continuous variables are presented as mean ± standard deviation (SD). Changes in temperature and scar-related variables over time were evaluated using repeated-measures analysis of variance and paired *t*-tests. Group comparisons of ΔT_2_ according to scar type (linear vs. hypertrophic) and anatomical location (face vs. extremities/trunk) were performed using independent *t*-tests. Pearson correlation coefficients were used to assess associations between ΔT_2_ and VSS score changes, and 95% confidence intervals were calculated for the correlation estimates. Linear regression was performed to identify predictors of scar improvement, including energy level, anatomical location, and ΔT_2_. Two-tailed *p*-values < 0.05 were considered statistically significant. No missing data were identified for primary outcomes.

## 3. Results

### 3.1. Patient Demographics and Baseline Characteristics

A total of 55 patients (21 males, 34 females) were included, with a mean age of 32.8 ± 9.4 years (range: 18–58 years). The mean duration of scars prior to treatment was 11.6 ± 4.2 months. Post-surgical scars were most common (n = 28, 50.9%), followed by traumatic scars (n = 18, 32.7%) and burn-related scars (n = 9, 16.4%). Anatomical distribution included the face (n = 22, 40.0%), extremities (n = 20, 36.4%), and trunk (n = 13, 23.6%).

According to Fitzpatrick skin classification, 32 patients (58.2%) were type III, and 23 (41.8%) were type IV. The baseline Vancouver Scar Scale (VSS) score averaged 6.3 ± 1.7. No significant differences were observed in baseline VSS scores or scar duration across scar etiologies or anatomical regions (*p* > 0.05). Table 1 summarizes baseline characteristics.

### 3.2. Thermal Response Analysis

Thermal imaging confirmed consistent post-laser temperature elevation. Baseline temperature prior to anesthetic application was 32.4 ± 0.9 °C (T_0_), with no significant change following anesthetic application (T_1_: 32.5 ± 0.8 °C, *p* = 0.41). Immediately after the treatment, mean temperature increased to 33.7 ± 0.7 °C (T_2_), producing a mean temperature change (ΔT_2_ = T_2_ − T_0_) of +1.3 ± 0.6 °C (*p* < 0.001).

**Figure 2:**
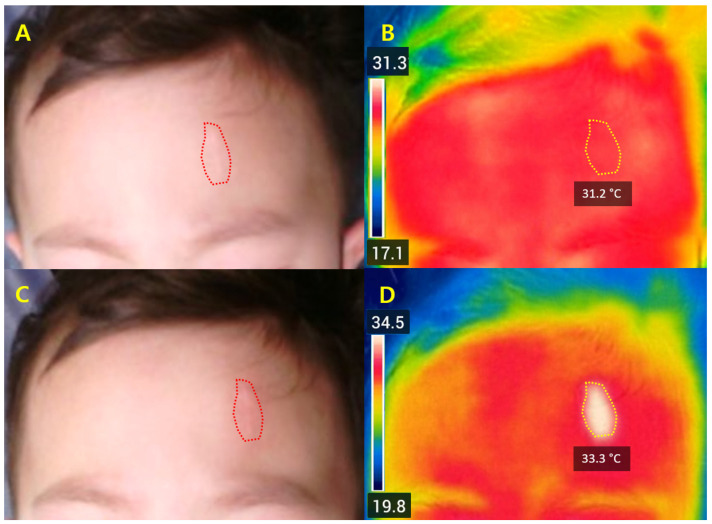
Representative FLIR thermal images obtained using the FLIR C5 camera before and immediately after Er:Glass laser treatment. (**A**,**B**) Pre-treatment photograph and corresponding thermal map (T_0_) showing a baseline temperature of 31.2 °C within the region of interest (ROI). (**C**,**D**) Post-treatment images (T_2_) demonstrate a localized temperature elevation to 33.3 °C (ΔT_2_ = +2.1 °C) restricted to the treated area, indicating effective dermal heating without diffuse thermal spread. The red dotted line in (**A**,**C**) denotes the scar location, and the yellow boundary in (**B**,**D**) marks the ROI for quantitative measurement.

Subgroup analysis revealed higher ΔT_2_ values in hypertrophic or thicker scars (+1.6 ± 0.5 °C) compared with thin linear scars (+1.0 ± 0.4 °C, *p* = 0.02). Temperature increases were also greater in scars on the extremities (+1.4 ± 0.5 °C) and trunk (+1.5 ± 0.4 °C) compared to facial lesions (+1.1 ± 0.6 °C, *p* = 0.04). Post hoc Bonferroni tests confirmed these differences. Results are presented in Table 2, with representative thermal maps shown in Figure 2.

### 3.3. Clinical Outcomes

At one-month follow-up, significant clinical improvement was observed. Mean VSS score decreased from 6.3 ± 1.7 to 4.2 ± 1.5 (*p* < 0.001), reflecting improved pigmentation, pliability, and texture. Mean scar area decreased by 23.5 ± 9.2% from baseline.

Pain during treatment was mild (VAS 2.1 ± 0.9), and discomfort resolved within 24 h. No delayed erythema, blistering, or other complications occurred. Patient satisfaction averaged 4.3 ± 0.6 on a 5-point Likert scale.

**Figure 3:**
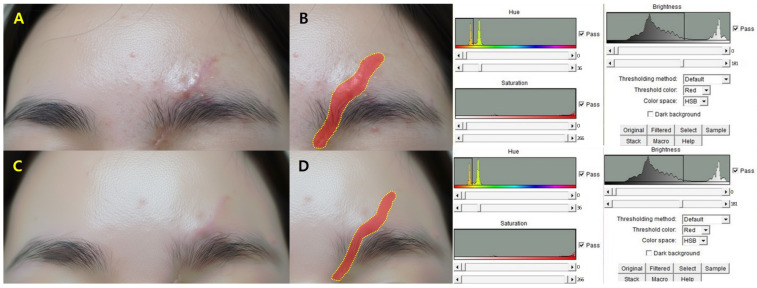
Early clinical improvement after non-ablative Er:Glass laser treatment. Representative images demonstrating both visual and quantitative changes one month post-treatment. (**A**,**B**) Baseline clinical image and ImageJ threshold-based area mapping of the hypertrophic scar. (**C**,**D**) At one month after Er:Glass laser treatment, notable improvement in color uniformity and surface contour is observed, with a reduction in threshold-defined scar area on planimetric analysis.

Patients with greater ΔT_2_ values (>1.5 °C) showed a larger mean VSS reduction (2.5 ± 1.0 points) than those with lower ΔT_2_ (<1.0 °C; 1.6 ± 0.8 points) (*p* = 0.03). Quantitative outcomes are shown in Table 3, with representative pre- and post-treatment images in Figure 3.

### 3.4. Correlation Between Thermal Dynamics and Clinical Improvement

**Figure 4:**
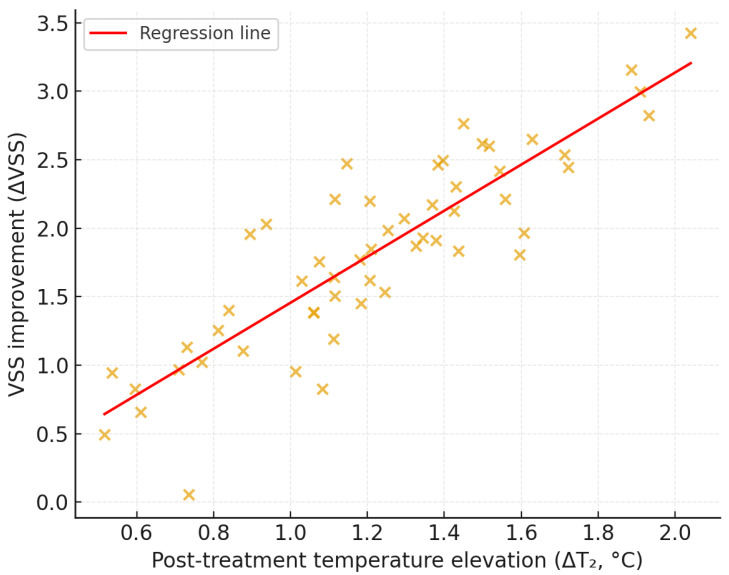
Positive correlation between post-treatment temperature elevation and scar improvement. Scatter plot illustrating the relationship between post-treatment temperature elevation (ΔT_2_) and improvement in Vancouver Scar Scale (VSS) score one month after Er:Glass laser treatment (*r* = 0.42, *p* = 0.003). The red line represents the fitted regression trend.

Post-treatment temperature elevation correlated positively with clinical improvement. ΔT_2_ correlated with VSS score reduction (*r* = 0.42, 95% CI 0.15–0.63, *p* = 0.003) and with scar-area reduction (*r* = 0.37, 95% CI 0.08–0.59, *p* = 0.02). In multivariate regression, ΔT_2_ remained an independent predictor of VSS improvement (β = 0.38, *p* = 0.01; 95% CI provided in Table 4) after adjusting for energy level and anatomical site. Treatment energy and anatomical region were not independently associated with outcome (*p* > 0.05). Results are summarized in Table 4, and the correlation trend is displayed in Figure 4. A supplementary histogram illustrating the distribution of ΔT_2_ values across the cohort is provided in Supplementary Figure S1 to further characterize the empirical basis of the exploratory thermal range.

### 3.5. Representative Case Demonstration

Three representative cases illustrate the clinical significance of ΔT_2_ in guiding effective treatment response.

**Figure 5:**
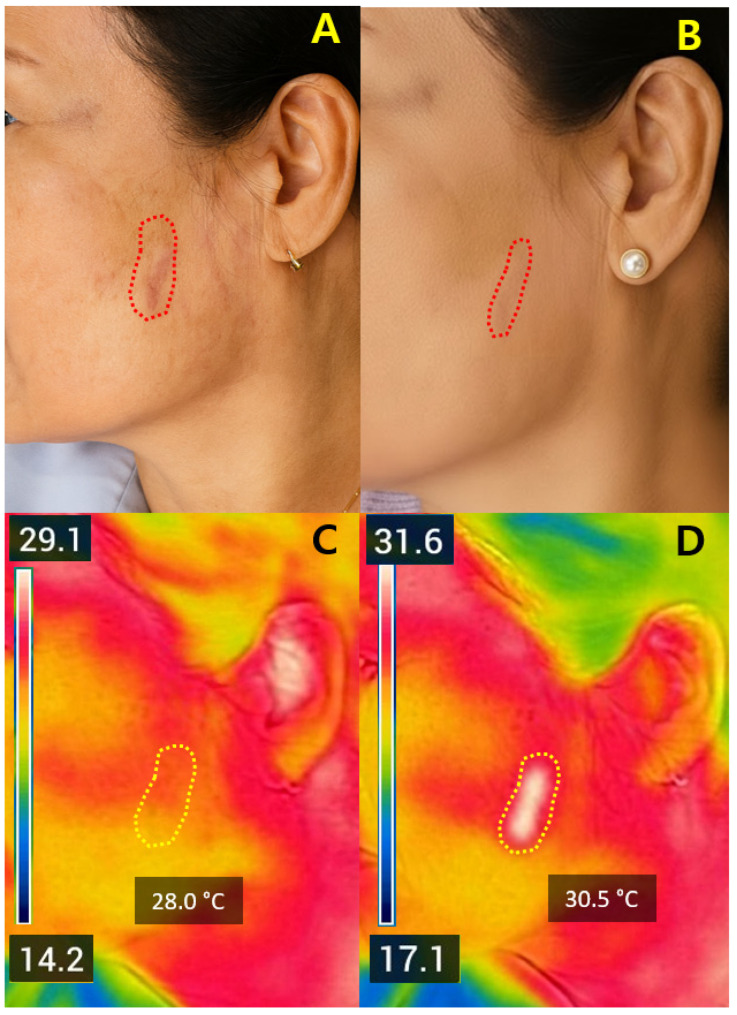
Early remodeling response of a post-surgical facial scar following non-ablative Er:Glass laser therapy. (**A**) Baseline clinical photograph of a 52-year-old woman showing a linear scar on the right cheek (red dotted line). (**B**) One-month follow-up image showing improved color uniformity and smoother surface texture without adverse effects. (**C**) Pre-treatment FLIR thermal map showing a baseline temperature of 28.0 °C within the treated region (yellow ROI). (**D**) Post-treatment thermal map showing a temperature elevation to 30.5 °C (ΔT_2_ = +2.5 °C) localized to the same ROI, indicating controlled dermal heating and early remodeling response. All photographs were obtained under routine clinical conditions; minor variations in lighting and positioning may occur due to the retrospective nature of image acquisition.

Case 1. A 52-year-old woman with a linear post-surgical cheek scar (baseline VSS = 5) treated at 2.0 mJ demonstrated a ΔT_2_ of +2.5 °C, with visible texture smoothing and color uniformity at one month and no complications (Figure 5).

**Figure 6:**
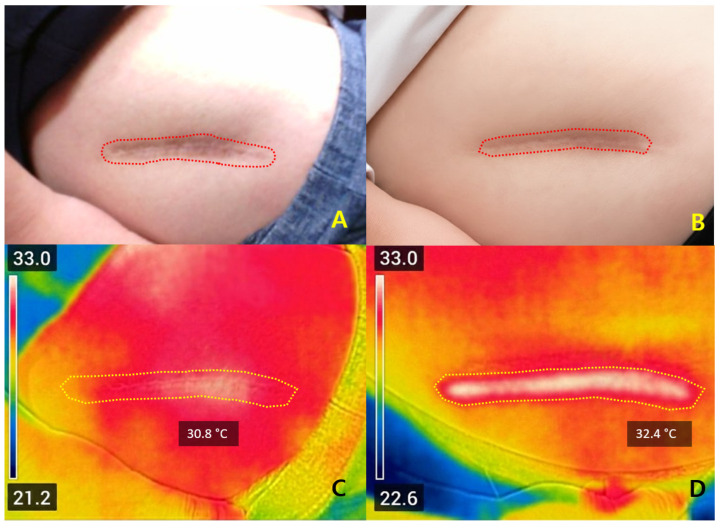
Early flattening of a hypertrophic post-surgical scar on the lateral thigh following Er:Glass laser treatment. (**A**) Baseline clinical photograph showing a hypertrophic linear scar on the lateral thigh (red dotted line). (**B**) One-month follow-up image demonstrating visible flattening and improved pigmentation, with no adverse effects observed. (**C**) Pre-treatment FLIR thermal map showing a baseline temperature of 30.8 °C within the treated region (yellow ROI). (**D**) Post-treatment thermal image showing a localized temperature elevation to 32.4 °C (ΔT_2_ = +1.6 °C), indicating controlled dermal heating consistent with sub-epidermal remodeling and early collagen reorganization. All photographs were obtained under routine clinical conditions; minor variations in lighting and positioning may occur due to the retrospective nature of image acquisition.

Case 2. A 36-year-old man with a hypertrophic thigh scar (baseline VSS = 7) treated at 3.5 mJ showed ΔT_2_ of +1.6 °C and flattening with VSS improvement to 4 at one month (Figure 6).

**Figure 7:**
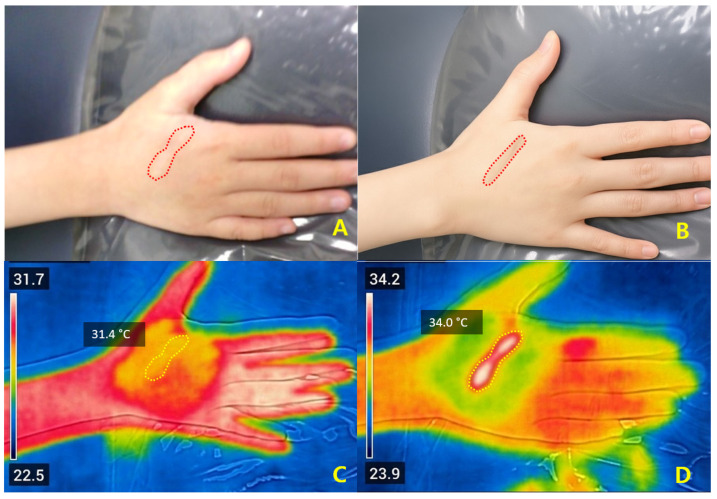
Early remodeling of a post-traumatic dorsal hand scar following Er:Glass laser treatment. (**A**) Baseline clinical photograph of a 42-year-old woman showing a linear post-traumatic scar on the right dorsal hand (red dotted line). (**B**) One-month follow-up image demonstrating smoother texture and more uniform coloration, with no adverse events observed. (**C**) Pre-treatment FLIR thermal map showing a baseline temperature of 31.4 °C within the treated region (yellow ROI). (**D**) Post-treatment thermal image demonstrating a localized temperature elevation to 34.0 °C (ΔT_2_ = +2.6 °C), indicating effective dermal heating within the upper safe range of the thermal window and subsequent collagen remodeling response. All photographs were obtained under routine clinical conditions; minor variations in lighting and positioning may occur due to the retrospective nature of image acquisition.

Case 3. A 42-year-old woman with a post-traumatic dorsal-hand scar (baseline VSS = 6) treated at 2.5 mJ demonstrated ΔT_2_ of +2.6 °C, with improved texture and color uniformity without adverse effects (Figure 7).

Collectively, these representative cases visually support the quantitative findings of this study, demonstrating that a moderate post-treatment temperature elevation (approximately 1.5–2.5 °C) corresponds to favorable early remodeling and improved scar appearance without complications.

These examples reinforce the clinical validity of ΔT_2_ as a safe and quantifiable indicator of optimal thermal response during non-ablative Er:Glass laser therapy.

### 3.6. Safety and Adverse Events

Procedures were well tolerated. Transient erythema occurred in 32.7% and mild edema in 21.8% of patients, resolving within 12–24 h without intervention. No blistering, infection, pigmentary changes, or delayed healing occurred. No patients required analgesics or topical steroids, and no treatment discontinuations occurred (VAS ≤ 3). Table 5 summarizes adverse events.

## 4. Discussion

This study suggested that non-ablative fractional Er:Glass laser treatment at 1550 nm was associated with early improvements in scar appearance, while real-time FLIR thermography was used to monitor surface thermal response during treatment. Quantitative thermal mapping showed heterogeneous post-treatment temperature elevations, and an exploratory range (approximately 1.5–2.5 °C) derived from representative cases was associated with early clinical improvements in scar texture, color uniformity, and Vancouver Scar Scale (VSS) scores. FLIR monitoring enabled objective visualization of surface thermal distribution without contact, offering immediate feedback on energy delivery [16]. FLIR thermography provides an indirect approximation of subsurface dermal heating rather than a direct measurement. Because surface temperature can be influenced by perfusion, epidermal cooling, and tissue optical properties, ΔT_2_ should be interpreted as an inferential indicator of thermal response rather than a precise quantification of intradermal heat. All patients recovered uneventfully without serious adverse events such as blistering or pigmentary change. Together, these findings suggest that ΔT_2_ may serve as a potential parameter reflecting surface thermal response during non-ablative fractional laser therapy and highlight the possible relevance of real-time thermal feedback as an exploratory monitoring framework. Although the mean ΔT_2_ across the cohort was 1.3 °C, this reflects an overall average that includes many scars with lower thermal responses; the proposed 1.5–2.5 °C range was therefore derived from individual cases demonstrating favorable early remodeling and should be interpreted as an exploratory trend rather than a definitive threshold. Importantly, the present data do not establish the 1.5–2.5 °C range as a therapeutic target, as no meaningful efficacy gradient within this interval could be identified.

Fractional laser systems are well recognized for improving scars through collagen remodeling and neocollagenesis [4,5]. While ablative fractional lasers (CO_2_, Er:YAG) provide pronounced textural improvement, they are often associated with prolonged erythema and downtime due to epidermal ablation [6,7]. By contrast, non-ablative systems—especially the 1550 nm Er:Glass laser—provide controlled sub-epidermal heating while preserving epidermal integrity, resulting in faster recovery and a lower risk of post-inflammatory pigmentation [8,19]. The current findings align with previous reports demonstrating improvements in scar pliability, vascularity, and pigmentation with minimal adverse events [11,19], but expand upon them by incorporating FLIR thermography to characterize thermal dynamics and provide physiological context for heating-induced remodeling. Recent clinical studies have also reported favorable outcomes of 1550 nm Er:Glass fractional photothermolysis for improving scar characteristics and dermal texture, particularly in atrophic and treatment-resistant lesions [20]. Our findings are consistent with these observations and extend them by providing quantitative thermal-response data obtained through real-time infrared imaging. This real-time approach addresses a persistent gap in the literature regarding standardized intra-treatment thermal monitoring [10,14,16]. The observed relationship between moderate ΔT_2_ elevation and early improvement indicates an exploratory thermal range that may be associated with safe collagen remodeling [10], providing preliminary support for temperature-informed monitoring rather than defining a fixed threshold [15,21]. The potential biological basis of this thermal response may relate to well-established heat-induced remodeling pathways: sub-epidermal heating can induce reversible collagen denaturation and contraction, trigger fibroblast activation, and promote subsequent neocollagenesis. Moderate thermal stress is also known to upregulate heat-shock proteins, which facilitate extracellular matrix repair and regulate collagen synthesis. In non-ablative fractional systems, microthermal zones created by controlled optical absorption and thermal diffusion act as focal points for dermal regeneration, enabling matrix turnover while sparing the epidermis [5]. However, FLIR thermography provides only an indirect approximation of subsurface dermal heating rather than a direct measurement. Surface temperature is influenced by multiple physiological variables—including blood flow, epidermal thickness, hydration, and local cooling—and therefore may not perfectly reflect the true intradermal thermal dose. Accordingly, the association between ΔT_2_ and remodeling should be interpreted as an inferential relationship, and future validation using ultrasonography, thermal modeling, or histologic correlation will be necessary to clarify the correspondence between surface and subsurface thermal behavior.

Table 6 situates the present findings within existing literature by comparing fractional laser systems. Non-ablative devices such as the 1550 nm Er:Glass laser achieve remodeling comparable to ablative systems but with shorter downtime and fewer complications [5,7,19].

These comparative data highlight the need for standardized definitions of downtime and objective recovery metrics [6,13]. Incorporating FLIR-based temperature elevation may provide a physiologic reference point to refine downtime classification, improve pre-treatment counseling, and support individualized energy titration, although its role remains exploratory [15,16].

The magnitude of dermal heating emerged as a key determinant of efficacy and safety. A consistent post-treatment temperature elevation (ΔT_2_ ≈ 1.5–2.5 °C) may represent an exploratory sub-epidermal thermal range potentially associated with collagen remodeling, while remaining below thresholds associated with epidermal protein denaturation [10,13,22]. Rather than relying solely on subjective clinical cues such as erythema or edema, ΔT_2_ measurement may offer a quantifiable reference point for estimating energy delivery in real time [4,13], although its predictive value remains limited [16].

FLIR-guided feedback also enables individualized energy titration according to scar thickness, anatomical site, and vascularity—factors known to influence optical absorption and thermal diffusion [4,23]. Instead of establishing ΔT_2_ as a biomarker, these findings suggest that it may function as a preliminary physiologic parameter to guide temperature-informed treatment adjustment, with further evidence required to determine its reproducibility across laser systems and operators [10,21]. Taken together, controlled temperature elevation may be associated with favorable remodeling, while maintaining epidermal integrity [4,22].

Because non-ablative fractional laser treatment produces minimal immediate visual changes during irradiation, it is often difficult to judge the adequacy of treatment by appearance alone. FLIR thermography complements this limitation by visualizing real-time thermal distribution, which may assist in achieving appropriate surface thermal response, though further validation is needed. In addition, the irradiation area (approximately 5–10 mm in diameter) can be adjusted according to individual scar morphology, enabling a personalized and reproducible treatment approach.

Real-time thermal imaging showed safe and localized surface temperature elevation. Baseline temperature increased from 32.4 ± 0.9 °C to 33.7 ± 0.7 °C immediately after irradiation (ΔT_2_ = +1.3 ± 0.6 °C, *p* < 0.001). Higher ΔT_2_ values were noted in thicker and hypertrophic scars (+1.6 ± 0.5 °C) versus thin linear scars (+1.0 ± 0.4 °C, *p* = 0.02), and in extremities/trunk compared with facial scars, reflecting anatomical differences in dermal thickness and perfusion [4,23,24,25]. A moderate temperature rise correlated with early clinical improvement (*r* = 0.42, *p* = 0.003), indicating an association rather than a predictive relationship [10,21,22]. Because topical anesthesia can transiently lower skin temperature, T_0_ was used as the baseline reference to isolate laser-associated temperature elevation and avoid confounding by anesthesia-related cooling. The observed association between ΔT_2_ and early improvement reflects short-term remodeling only, and further long-term follow-up is required to determine whether these early changes translate into sustained clinical benefit.

Representative cases illustrated that ΔT_2_ values between +1.4 °C and +2.6 °C were associated with favorable early improvement without complications. Safety outcomes again support that ΔT_2_ within this moderate range may be compatible with adequate dermal stimulation without apparent complications, while preventing excessive injury [10,16,22]. Compared with ablative systems—typically requiring 5–10 days of downtime—Er:Glass treatment achieved remodeling with only 1–3 days of recovery and minimal risk of post-inflammatory hyperpigmentation [5,7,19]. FLIR feedback may help maintain energy delivery within a provisional thermal range, but further prospective validation is warranted [15,16]. Although these findings do not establish a definitive threshold, they suggest that ΔT_2_ within the 1.5–2.5 °C range may serve as an exploratory indicator of favorable early response. FLIR-assisted Er:Glass therapy may therefore offer a preliminary framework for temperature-informed monitoring, although further validation is required.

Several limitations merit consideration. Given the retrospective, single-center design and the absence of a control group, the evidence provided by this study should be regarded as preliminary and hypothesis-generating rather than confirmatory, and the findings must therefore be interpreted with appropriate caution. This retrospective single-center study may introduce selection bias and limit generalizability, although standardized treatment and imaging protocols enhance internal validity. Importantly, the retrospective design relied on non-standardized treatment parameters, including variations in energy settings, pass numbers, and density, all of which may act as potential confounding factors. Because of these methodological constraints and the absence of a control group, the association observed between post-treatment temperature elevation and early clinical improvement cannot be interpreted as causal. Consequently, the proposed thermal window should be regarded as exploratory rather than definitive. The one-month follow-up focused on early remodeling; longer-term evaluation over 6–12 months is necessary to determine sustained improvement. Given this limited follow-up period, the durability of collagen remodeling and the persistence of clinical improvement remain unknown, and the predictive value of ΔT_2_ for long-term outcomes cannot be concluded from the present data. Because scar maturation occurs over several months, the present findings cannot determine whether the early ΔT_2_–response relationship persists over 3, 6, or 12 months. Thus, the long-term predictive value of ΔT_2_ cannot be determined from the present dataset. FLIR thermography measures surface temperature and does not directly reflect subsurface heating; however, prior optical modeling suggests a potential relationship between surface and dermal thermal behavior. As FLIR measures only surface temperature, the correspondence between ΔT_2_ and true intradermal heating remains indirect and requires further validation. Additionally, thermal image acquisition and ROI placement—although performed by a single operator to ensure consistency—may still be subject to operator dependence, which could introduce measurement variability. Topical anesthesia may also transiently alter surface temperature through cooling or occlusive effects, potentially affecting pre-treatment baseline values despite our use of T_0_ to minimize this source of bias. Future work incorporating histological or ultrasonographic validation would strengthen these observations [26]. The absence of a control group limits direct comparison across energy settings or systems; future prospective studies with randomized controlled designs, predefined laser parameters, and comparison groups will be essential to validate whether maintaining a target ΔT_2_ truly leads to superior and durable clinical outcomes. Multicenter investigations incorporating standardized protocols will further improve generalizability and help establish ΔT_2_ as a potential treatment-guiding parameter rather than a fully validated biomarker. Lastly, representative clinical photographs were obtained from routine clinical records and therefore exhibit natural variability in lighting, positioning, and scale, which may limit the objectivity of visual comparison across time points.

## 5. Conclusions

This study suggests that non-ablative fractional Er:Glass laser therapy, when combined with real-time FLIR thermography, may offer a potential approach for monitoring surface thermal response during scar treatment. However, a moderate post-treatment temperature increase (ΔT_2_ ≈ 1.5–2.5 °C) should be interpreted as an exploratory finding rather than a definitive therapeutic threshold. The observed association between ΔT_2_ and early clinical improvement is preliminary and does not establish a causal relationship. Accordingly, ΔT_2_ may serve as a potential physiologic parameter that is associated with favorable early response, rather than a validated biomarker of treatment adequacy. Further prospective, controlled, and long-term studies will be required to determine whether temperature-based monitoring has clinical relevance for durable remodeling and to clarify the role of thermal assessment in future treatment evaluation.

## Figures and Tables

**Table 1:** Patient demographics and baseline clinical characteristics.

Variable	Value	
Age (years)	Mean ± SD (range)	32.8 ± 9.4 (18–58)
Sex	Male/Female	21 (38.2%)/34 (61.8%)
Scar duration (months)	Mean ± SD	11.6 ± 4.2
Etiology	Surgical	28 (50.9%)
	Traumatic	18 (32.7%)
	Burn	9 (16.4%)
Anatomical location	Face	22 (40.0%)
	Extremities	20 (36.4%)
	Trunk	13 (23.6%)
Fitzpatrick skin type	Type III	32 (58.2%)
	Type IV	23 (41.8%)
Baseline VSS score	Mean ± SD	6.3 ± 1.7

Note: Data are presented as mean ± standard deviation or number (%). No significant intergroup differences were observed (*p* > 0.05).

**Table 2:** Thermal response parameters recorded before and after Er:Glass laser treatment.

Parameter	Category	T_0_ (°C)	T_1_ (°C)	T_2_ (°C)	ΔT_2_ (°C, Mean ± SD)	*p*-Value
Overall (n = 55)	—	32.4 ± 0.9	32.5 ± 0.8	33.7 ± 0.7	+1.3 ± 0.6	<0.001
Scar type	Linear (n = 28)	32.3 ± 0.8	32.4 ± 0.8	33.3 ± 0.7	+1.0 ± 0.4	—
	Hypertrophic (n = 27)	32.5 ± 0.9	32.6 ± 0.8	34.1 ± 0.6	+1.6 ± 0.5	0.02
Anatomical location	Face (n = 22)	32.5 ± 0.9	32.6 ± 0.7	33.6 ± 0.8	+1.1 ± 0.6	—
	Extremities (n = 20)	32.4 ± 0.8	32.5 ± 0.8	33.8 ± 0.6	+1.4 ± 0.5	0.04
	Trunk (n = 13)	32.3 ± 1.0	32.5 ± 0.9	33.8 ± 0.7	+1.5 ± 0.4	0.04

Note: Within-group changes (T_2_ vs. T_0_) were analyzed using paired *t*-tests. Between-group comparisons of ΔT_2_ across scar types and anatomical locations were performed using independent *t*-tests or one-way ANOVA as appropriate.

**Table 3:** Clinical outcomes one month after erbium glass laser treatment.

Parameter	Baseline	1 Month	Change (Δ)	*p*-Value
VSS score	6.3 ± 1.7	4.2 ± 1.5	−2.1 ± 1.0	<0.001
Scar area (cm^2^)	4.8 ± 2.1	3.7 ± 1.9	−23.5 ± 9.2%	<0.01
VAS pain	—	2.1 ± 0.9	—	—
Patient satisfaction (5-point Likert)	—	4.3 ± 0.6	—	—

Note: Data are expressed as mean ± SD. *p*-values were obtained from paired *t*-tests comparing the baseline and 1-month values, where applicable.

**Table 4:** Correlation and multivariate regression analysis of ΔT_2_ with clinical improvement parameters.

Variable	Pearson *r* (95% CI)	*p*-Value	Regression β	Adjusted *p*-Value
ΔT_2_ vs. VSS change	0.42 (0.15–0.63)	0.003	0.38	0.01
ΔT_2_ vs. scar area reduction	0.37 (0.08–0.59)	0.02	0.33	0.04
Energy level vs. VSS change	0.12	0.34	—	—
Site (face/extremity/trunk) vs. VSS change	—	0.27	—	—

Note: *p*-values from Pearson’s correlation; adjusted *p*-values from the linear regression model.

**Table 5:** Summary of procedure-related adverse events following non-ablative fractional Er:Glass laser treatment.

Adverse Event	Incidence (n, %)	Duration (h)	Additional Management
Transient erythema	18 (32.7%)	12–24	None
Mild edema	12 (21.8%)	12–24	None
Blistering	0 (0%)	—	—
Infection	0 (0%)	—	—
Pigmentary alteration	0 (0%)	—	—
Delayed wound healing	0 (0%)	—	—

**Table 6:** Comparative characteristics of commonly used fractional laser systems for scar treatment.

Laser Type	Wavelength (nm)	Ablation Type	Penetration Depth	Typical Energy (mJ)	Endpoint Parameter	Expected Downtime	Common Adverse Effects
CO_2_ (Fractional)	10,600	Ablative	200–2000 µm	30–100	Erythema, pinpoint bleeding	5–10 days	Crusting, PIH, prolonged redness
Er:YAG (Fractional)	2940	Ablative	100–400 µm	5–50	Desquamation, erythema	3–7 days	Dryness, burning, PIH
Er:Glass	1550	Non-ablative	1000–1500 µm	2–4	Mild edema, transient erythema	1–3 days	PIH (rare), warmth, tenderness
Nd:YAG	1320	Non-ablative	1000–1500 µm	20–50	Mild erythema, swelling	<2 days	Edema, PIH (rare)
Diode	1450	Non-ablative	800–1200 µm	10–30	Minimal edema	1–2 days	Dryness, rare PIH

Note: Data were derived from previously published literature and manufacturer specifications [5,7,19]. Downtime and adverse events represent approximate clinical ranges. PIH, post-inflammatory hyperpigmentation.

## Data Availability

The data presented in this study are available on request from the corresponding author due to restrictions related to patient privacy and institutional ethical guidelines.

## Supplementary Materials

The following supporting information can be downloaded at: https://www.mdpi.com/article/10.3390/jcm14248910/s1, Figure S1. Histogram showing the distribution of post-treatment temperature elevation (ΔT_2_) across the cohort (n = 55). The y-axis represents the number of patients within each ΔT_2_ interval. Mean and median ΔT_2_ values are indicated, and the shaded region (1.5–2.5 °C) represents the exploratory thermal window derived from individual cases demonstrating favorable early improvement.

## Abbreviations

The following abbreviations are used in this manuscript:

CO_2_	Carbon dioxide
Er:YAG	Erbium-doped yttrium aluminum garnet
Er:Glass	Erbium-doped glass
FLIR	Forward-looking infrared
ROI	Region of interest
VSS	Vancouver Scar Scale
VAS	Visual Analog Scale
Nd:YAG	Neodymium-doped yttrium aluminum garnet
PIH	Post-inflammatory hyperpigmentation
