# Supplementary material for: Thermal Response-Based Evaluation of Non-Ablative Fractional Er:Glass Laser Therapy for Scar Management: A Retrospective Observational Study with Forward-Looking Infrared (FLIR) Monitoring
Source: J Clin Med. 2025 Dec 17;14(24):8910. doi: 10.3390/jcm14248910 (PMC12734238; doi:10.3390/jcm14248910)
Supplement: Supplementary file 1 [file jcm-14-08910-s001.zip › jcm-4004798-supplementary.docx]

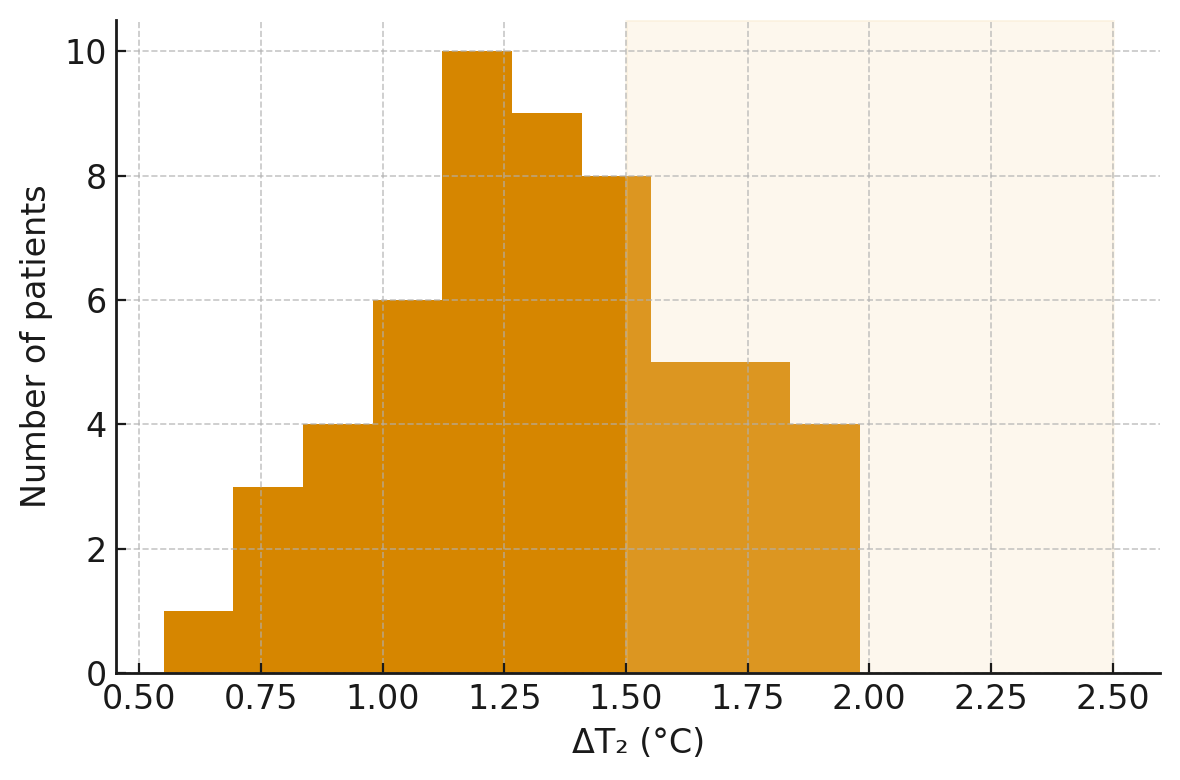


**Supplementary Figure S1.** Histogram showing the distribution of post-treatment temperature elevation (ΔT₂) across the cohort (n = 55). The y-axis represents the number of patients within each ΔT₂ interval. Mean and median ΔT₂ values are indicated, and the shaded region (1.5–2.5 °C) represents the exploratory thermal window derived from individual cases demonstrating favorable early improvement.
